# A Latent Profile Analysis of Latino Adolescents’ Cultural Wealth, Ethnic-Racial Discrimination, and Academic Adjustment

**DOI:** 10.1007/s10964-026-02328-7

**Published:** 2026-02-19

**Authors:** Stefanie Martinez-Fuentes, Adriana J. Umaña-Taylor, Kimberly A. Updegraff, Deborah Rivas-Drake, David R. Schaefer, Allison M. Ryan, Katharine H. Zeiders

**Affiliations:** 1 University of Illinois Urbana-Champaign, Urbana, IL USA; 2 Harvard University, Cambridge, MA USA; 3 Purdue University, West Lafayette, IN USA; 4 University of Michigan, Ann Arbor, MI USA; 5 University of California Irvine, Irvine, CA USA; 6 University of Arizona, Tucson, AZ USA

**Keywords:** Latino, Ethnoracial discrimination, Academic adjustment, Friendships, Ethnoracial socialization, Ethnic-racial identity

## Abstract

Drawing from the model of community cultural wealth, which suggests that youth from minoritized populations can draw on their cultural knowledge, networks, and skills to successfully navigate their academic contexts, the current study examined whether latent profiles of cultural capital emerged among a sample of Latino adolescents using various indicators of cultural wealth. The sample included 627 Latino adolescents (95% U.S.-born; 57% girls; 33% 9th graders, 27% 10th graders, 23% 11th graders, 18% 12th graders). Specifically, configurations of cultural wealth were analyzed based on the following dimensions: familial ethnic socialization, ethnic-racial identity components, and emotional support from Latino and non-Latino friendship networks. Research questions also included testing whether profiles characterized by greater cultural wealth exhibited better academic adjustment (i.e., emotional and behavioral academic adjustment, academic self-efficacy, and school belonging), and whether the association between ethnoracial discrimination and academic adjustment was moderated by profile membership. Findings revealed four profiles, with the largest (38%) consisting of adolescents with high cultural wealth (i.e., *Achieved*,* High FES*,* High Friend Support*). Furthermore, this profile exhibited better academic adjustment relative to the profiles that varied in levels of cultural wealth. When examining whether profiles with more cultural wealth also demonstrated better academic adjustment in the face of ethnoracial discrimination, findings revealed that a subgroup with more moderate cultural wealth (*Moratorium*,* Moderate FES*,* High Friend Support*) was more vulnerable to discrimination for most indicators of adjustment compared to all other profiles. Overall, findings extend previous variable-centered work by showing that Latino adolescents have heterogeneous experiences with cultural wealth, and examining these subgroups of adolescents provides novel insights into understanding how cultural wealth differently relates to adolescents’ academic adjustment, including in the context of ethnoracial discrimination.

**Supplementary Information:**

The online version contains supplementary material available at 10.1007/s10964-026-02328-7.

## Introduction

Since 2013, Latino youth have exhibited a trend toward greater academic achievements than ever before, including lower high school dropout rates and greater college enrollment rates (National Center for Education Statistics, 2024). However, academic disparities persist, as Latino youth continue to drop out of high school more frequently and have lower college enrollment rates compared to their peers from other ethnoracial groups (National Center for Education Statistics, 2024). These ongoing disparities in educational attainment make it imperative to understand the resources that can support Latino adolescents’ academic success and to find solutions to the barriers that prevent them from reaching their academic potential. To explore potential cultural assets, the current study drew from a community cultural wealth framework (Yosso, 2005) to assess the salience of various indicators of cultural wealth, including: family ethnic socialization, ethnic-racial identity, and emotional support from Latino and non-Latino friends. Furthermore, based on a risk and resilience framework (Masten & Barnes, 2018), the current study examined whether configurations of cultural wealth could promote adolescents’ academic adjustment and buffer the impact of their experiences with discrimination.

## Latino Adolescents’ Academic Adjustment and Ethnoracial Discrimination

Within the academic setting, there are multiple factors that reflect adolescents’ capacity to perform well in school and are related to their future academic potential. For instance, behavioral (i.e., active participation) and emotional (i.e., positive classroom experiences) engagement, which capture two distinct forms of student participation, are each positively linked with students’ academic performance and educational aspirations (Wang & Eccles, 2012). In addition, adolescents’ academic self-efficacy, or feeling confident about their ability to do well in school despite challenges, reflects students’ resilience within their school setting and has been positively associated with Latino adolescents’ grade point averages (Manzano-Sanchez et al., 2018). Furthermore, school belonging is indicative of students’ perceptions that they are being treated fairly in school, which may in turn facilitate their learning (Korpershoek et al., 2020). Notably, there is evidence that academic outcomes can vary by youth sociodemographic characteristics, including their gender and grade level. For instance, girls tend to report more favorable academic adjustment compared to boys, and changes in academic outcomes have also emerged across grade levels (Brass et al., 2019; Cupito et al., 2015; Evans et al., 2018). Thus, when assessing predictors of academic adjustment, it is important to account for these sociodemographic characteristics.

When examining the academic adjustment of Latino adolescents, it is also necessary to consider other factors that may hinder their ability to reach their academic potential (García Coll et al., 1996). Specifically, U.S. Latino adolescents are developing within a system of ethnoracial stratification that has historically placed individuals from ethnoracially minoritized groups toward the bottom of the social hierarchy, which in turn is expected to have negative implications for adolescents’ academic adjustment (García Coll et al., 1996). Adolescence is a critical developmental period to examine youth’s experiences with systems of social stratification, as this period is marked by their enhanced skills to engage in more abstract thinking, including an understanding of social categorization based on ethnicity and race and the potential disadvantages they may be subjected to because of their ethnoracial group membership (Umaña-Taylor, 2016a). Furthermore, evidence suggests that experiences of ethnoracial discrimination in adolescence are more harmful for one’s later physical and psychological health compared to experiences in young adulthood (e.g., Adam et al., 2015).

Prior research suggests that Latino adolescents are aware that adults and peers may have negative perceptions of them and of their ethnoracial group as a whole (Constante et al., 2021; Zeiders et al., 2021), and this may be particularly the case for adolescents who reside in geographic areas characterized by persistent anti-immigrant policies, practices, and sentiments that target Latinos (Ayón, 2016), such as Arizona, the location of the current study. Prior research has linked Latino adolescents’ frequent experiences of ethnoracial discrimination with lower feelings of school belonging and less academic engagement (Gonzalez et al., 2014; Martinez-Fuentes et al., 2021). However, Latino adolescents may also have access to resources that function as a “backup system,” allowing them to adapt to their environment in a manner that may counteract the risk of discrimination on their academic success (Masten & Barnes, 2018).

### Latino Adolescents’ Sources of Community Cultural Wealth

The community cultural wealth model describes familial (e.g., community within families), navigational (e.g., “inner resources” that facilitate individuals’ abilities to thrive in the face of risk), and social (e.g., emotional support from close relationships) capital as sources of cultural wealth that can collectively inform adolescents’ positive academic adjustment (Yosso, 2005). Drawing from this model, familial capital is a dimension of cultural wealth that encompasses learning about one’s ethnoracial background, which may promote a sense of familial bonding that helps adolescents feel connected to their cultural community and navigate stressful experiences (Yosso, 2005). Thus, the current study focused on *familial ethnic socialization*, or FES, as a form of familial capital. Although there are other ethnoracial socialization strategies, FES refers to the overt (e.g., family members teaching adolescents about their cultural history) and covert (e.g., spending time with people who share one’s ethnoracial background) messages that teach adolescents about their ethnoracial background (Umaña-Taylor et al., 2004). Prior work suggests that FES experiences may provide adolescents with a sense of community that instills feelings of cultural pride as well as a positive self-perception that enhances their ability to succeed academically (Wang et al., 2020). The protective effects of FES have also been demonstrated in prior empirical work, which has suggested that adolescents who experience discrimination may still demonstrate high levels of school engagement in the context of high FES (Chen et al., 2020).

Adolescence is also a period in which youth engage in the process of identity formation, which may underlie navigational capital for ethnoracially minoritized youth. In particular, the current study focused on the ethnic-racial identity (ERI) dimensions of *exploration* (i.e., seeking information that helps adolescents learn about their ethnic-racial group) and *resolution* (i.e., having a clear sense of what their ERI means to them; Umaña-Taylor, 2016b). The focus on these components of youth’s ERI derives from Erikson’s psychosocial development theory (1968), which posited that adolescence is the developmental stage during which youth are tasked with coming to a sense of understanding about who they are and their place in society, and this clarity ideally stems from one’s own search, or exploration (Erikson, 1968). Further, youth who are unable to reach this sense of clarity through personal exploration may be subjected to identity confusion (Erikson, 1968). To facilitate measuring the progression of identity development, Marcia (1980) operationalized Erikson’s theoretical notions of exploration and resolution (i.e., commitment) into a typology that consisted of four identity statuses. Specifically, an “achieved” identity status was the result of having reached a relatively high level of clarity about one’s identity through one’s self-directed exploration. In contrast, a “diffused” status reflected individuals in a state of identity confusion due to a lack of identity exploration and low resolution regarding their sense of self. Marcia (1980) also described two intermediary statuses, including “moratorium,” which captured a possible sense of identity rumination due to engaging in relatively higher levels of exploration but exhibiting low clarity. Finally, a “foreclosed” status captured a relatively higher level of resolution that was attained with minimal exploration, which was posited to result from the internalization of the values and commitments imposed by others (Marcia, 1980). This work did not explicitly focus on *ethnic-racial identity* development; however, Phinney (1993) extended this theorizing to ethnic identity development and subsequent work on ethnic-racial identity applied Marcia’s identity status framework to describe and measure the process by which adolescents explore and develop a sense of clarity regarding the aspects of their identity derived from their cultural, ethnic, and racial backgrounds (Phinney & Ong, 2007; Umaña-Taylor et al., 2004). Drawing from this conceptual work, it is posited that adolescents in an achieved ERI status will exhibit relatively better adjustment and be more psychologically protected in the face of ethnoracial discrimination, given that this status is characterized by a sense of confidence and self-assuredness that has been informed by individuals’ personal exploration and reflection about who they are (Umaña-Taylor, 2016b). These assertions have been supported empirically, as studies have found that those with an achieved ERI status demonstrated better academic outcomes compared to adolescents in less developed statuses (e.g., Miller-Cotto & Byrnes, 2016; Wantchekon & Umaña-Taylor, 2021). ERI achievement has also been positively linked with academic engagement after accounting for ethnoracial discrimination (Martinez-Fuentes et al., 2021).

Given that forming supportive friendships is another key developmental task during adolescence (Brown & Larson, 2009) and may enhance or compensate for support received from families (e.g., Yosso, 2005), the current study also considered the role of *peer emotional support* as a type of social capital that could inform adolescents’ academic adjustment. Specifically, the current study builds on a growing body of research assessing the role of friendships among ethnoracially minoritized adolescents and implications for their adjustment by differentiating support from friends who share a cultural background and those who do not (e.g., Benner & Wang, 2017). For instance, the academic implications of friendships between Latino adolescents and their Latino peers have received limited attention, although it is posited that emotional support from these friendships may serve a unique role that contributes to positive adjustment. Latino adolescents learn acceptable behaviors and norms from family members and, as a result, Latino adolescents may be drawn to form friendships with peers who share a cultural background (Mushonga & Henneberger, 2019). Indeed, one study with Mexican-origin families revealed that congruence across parent-peer contexts was associated with more positive adjustment (Updegraff et al., 2006). Although prior work has examined the association between Latino adolescents’ friendships and their academic adjustment (e.g., Delgado et al., 2016), it is unclear whether the friendships captured were between Latino adolescents and their Latino peers. Thus, the current study included this culturally specific network of friends as a potential resource that Latino adolescents may rely on to support their academic journey and protect against ethnoracial discrimination experiences.

Furthermore, intergroup contact theory posits that increased contact between individuals from different social groups can foster greater understanding and lead to reduced prejudice or preconceived notions individuals may have about different groups (Allport, 1954). Previous work found that having positive relationships with peers who are outside of one’s cultural group served a promotive role for adolescents’ academic adjustment and protected youth from the harmful consequences of ethnoracial discrimination (Benner & Wang, 2017). Overall, findings suggest that the friendships Latino adolescents establish with their non-Latino peers may provide adolescents with a greater sense of confidence in navigating relationships with people who do not share their ethnoracial background. Thus, the support from this particular type of friend group may be a form of cultural wealth that enables adolescents to succeed academically in the face of risk (Mushonga & Henneberger, 2019).

### Latent Profile Analysis for the Study of Latino Adolescents’ Cultural Wealth

The aforementioned work has predominantly examined the independent or unique associations between Latino adolescents’ cultural wealth and academic outcomes, and such an approach assumes that these resources exist in isolation from one another. Using a person-centered approach, the current study extends prior work by empirically examining whether subgroups that reflect different profiles of cultural wealth exist among Latino adolescents. In addition to a profile of adolescents who exhibit high levels of cultural wealth and another profile reflecting adolescents who experience low levels across all sources, it is possible that a third constellation captures adolescents who are relatively more “peer-centered,” such that emotional support from Latino and non-Latino friends are particularly salient in their lives relative to FES and ERI. Findings can illuminate the most common profiles of FES, peer support, and ERI, and whether particular constellations are linked to more positive academic adjustment and mitigate the effects of ethnoracial discrimination, which in turn can be informative for future work and policies focused on promoting Latino adolescents’ academic outcomes.

## Current Study

Prior work has primarily used variable-centered approaches to study how different forms of cultural wealth inform Latino adolescents’ academic adjustment and mitigates ethnic-racial discrimination. However, this approach masks potential heterogeneity in Latino youth’s experiences with various sources of cultural wealth and in turn how these different experiences uniquely inform their academic outcomes. Drawing upon a model of community cultural wealth, the current study used a person-centered approach to examine the co-occurrence (patterns) of FES, ERI exploration, ERI resolution, and emotional support from Latino and non-Latino friends, and whether profiles of cultural wealth indicators mitigated the negative impact of ethnoracial discrimination on Latino adolescents’ academic adjustment. Although the number of profiles is exploratory, it is hypothesized that patterns will emerge that are comprised of uniformly high and uniformly low levels on all resources. Other emergent profiles may represent variability in cultural wealth (Hypothesis 1). Adolescents with uniformly high levels of cultural wealth were hypothesized to report the most positive academic adjustment (Hypothesis 2). Finally, the association between ethnoracial discrimination and academic adjustment was expected to be moderated by profile membership, such that a weaker or nonsignificant association would emerge for adolescents in profiles characterized by high levels of cultural wealth, relative to adolescents in profiles characterized by lower or moderate levels of cultural wealth (Hypothesis 3).

## Method

### Participants and Procedures

Data were drawn from the last two waves of a three-wave longitudinal study assessing the role of peer networks on adolescents’ ERI development in the southwestern and midwestern U.S. In the present study, only data from the Southwestern site were used given that the sociohistorical context of the state where data were gathered (i.e., Arizona) may make Latino adolescents particularly susceptible to experiencing ethnoracial discrimination (Zeiders et al., 2021) and the relatively small sample size of Latino students in the Midwestern site (*n* = 99) precluded an examination by regional context. Survey data collection began in Spring 2017, followed by Fall 2018, and Spring 2018. The ethnoracial composition of the high school where data were collected was such that no single group was an ethnoracial majority (> 50%): 32% Hispanic/Latino, 31% White, 24% African American, 5% Multiple races, 4% Native American, 2% Asian, and less than 2% Pacific Islander (Arizona Department of Education, 2018).

To capture students’ experiences across an academic year, the current study focused on Waves 2 and 3 (hereafter referred to as T1 and T2). Only adolescents who self-identified as Latino/Hispanic (*n* = 636) at T1 were considered for the study. Given that 9 students were missing data on all study variables, and thus were excluded from all analyses, the analytic sample included 627 Latino adolescents. Most adolescents were U.S.-born (*n* = 597; 95.2%). Adolescents identified as girls (57.3%), boys (40.7%), or another gender (0.2%); the remaining were missing (1%). Participants included ninth graders (32.5%), tenth graders (26.3%), eleventh graders (22.5%), and twelfth graders (17.7%) with 1% missing grade level. Each adolescent was asked to provide their country of birth, as well as the birth country for their mother, father, and maternal and paternal grandparents. Among those who had available information for all family members (*n* = 515; 82% of the sample), 3% were first generation (i.e., all family members were born outside of the U.S.), 31% were second generation (i.e., U.S.-born adolescent, all family members were foreign-born), 18% were 2.5 generation (i.e., U.S.-born adolescent, one parent foreign-born and one parent U.S.-born), 26% were third generation (i.e., U.S.-born adolescent and U.S.-born mother and father, at least one foreign-born grandparent), and 22% were fourth generation (i.e., all family members were U.S.-born). Among adolescents who reported their mothers’ highest education level (*n* = 543; 86.6% of the sample), 18% had lower than a high school degree, 25.4% received a high school degree or equivalent, and 56.5% attained at least a bachelor’s degree. Among adolescents who reported their fathers’ highest education level (*n* = 524; 83.6% of the sample), 26.3% had fathers with lower than a high school degree, 32.1% received a high school degree or equivalent, and 41.8% attained at least a bachelor’s degree.

Research team members distributed paper and pencil surveys to teachers, who administered surveys during regular school hours to all students. The survey was only available in English. A passive consent process with an “opt-out” option was used for the survey; opt-out consent forms were delivered to the school and distributed by teachers for participants to share with their parent/guardian. Prior to completing the survey, adolescent participants were asked to provide assent. Approximately 82% of participants completed surveys. The institutional review boards at the participating university and school district approved the study.

### Measures

#### Family ethnic socialization (T1)

Participants completed the 12-item Familial Ethnic Socialization Measure (FESM) to assess the degree to which they had learned about their cultural background through family interactions, conversations, and activities (Umaña-Taylor et al., 2004). Items (e.g., “My family teaches me about our family’s ethnic/cultural background”) were scored on a 5-point Likert scale (1 = *not at all* to 5 = *very much*). A mean score of all items was computed and higher scores indicated higher levels of familial ethnic socialization. The FESM has been used with Latino youth samples (e.g., Santiago et al., 2016) and Cronbach’s α in the current study was 0.94.

#### Ethnic-racial identity (T1)

Adolescents completed the exploration and resolution subscales of the Ethnic Identity Scale-Brief (EIS-B; Douglass & Umaña-Taylor, 2015a) measure. The exploration (e.g., “I have attended events that have helped me learn more about my ethnicity”) and resolution subscales (e.g., “I have a clear sense of what my ethnicity means to me”) each consisted of three items. Items were scored on a 4-point Likert scale (1 = *does not describe me at all* to 4 = *describes me very well)* and were averaged to create each scale score, such that higher scores indicated greater exploration and resolution (i.e., clarity). Support for acceptable reliability and validity of EIS-B subscales with Latino adolescents has been demonstrated in prior work (e.g., Sanchez et al., 2017); in the current study, Cronbach’s α for exploration was 0.83, and for resolution was 0.86.

#### Friend emotional support (T1)

Friendship networks were assessed by asking adolescents to nominate up to ten of their closest friends in school. For each friend that was nominated, participants were asked to complete the emotional support scale from the Network of Relationships Inventory (Buhrmester & Furman, 2008), which consists of three items (e.g., “How often do you turn to this person for support with personal problems?”) scored on a 5-point Likert scale (1 = *never or hardly at all* to 5 = *always or extremely much*). To differentiate between emotional support from Latino and non-Latino friends (i.e., any ethnoracial group other than Latino), friend groups were created based on nominated friends’ self-reported ethnoracial group membership. A mean score of emotional support was computed for each friend individually based on target participants’ responses to the three items for each friend. Friends’ reports were categorized as Latino and non-Latino. An overall mean score was then computed for the target adolescents’ network of their Latino friends and their non-Latino friends and higher scores reflected more emotional support from each friend network. If target participants did not nominate friends for either friendship network, their emotional support scale was coded as missing for that specific friendship network. The subscale has demonstrated adequate reliability and validity in prior work with Latino youth (e.g., Niwa et al., 2014). Cronbach’s α for Latino friends was 0.93 and for non-Latino friends was 0.90.

#### Ethnoracial discrimination (T1) 

Adolescents were asked to report how frequently they had been mistreated because of their race or ethnicity using a modified version of the Adolescent Discrimination Distress Index (Fisher et al., 2000). The 11-item scale assessed discrimination from peers (e.g., “Were you called insulting names by other kids because of your race/ethnicity?”; 4 items), adults in school (e.g., “Were you put in a lower ability class or group because of your race/ethnicity?”; 3 items), and societal or institutional sources (e.g., “Were you hassled by the police because of your race/ethnicity?”; 4 items) over the past year. Modifications included expanding the response option from yes or no to a 5-point Likert scale (1 = *never* to 5 = *a whole lot*). Items were re-worded to specifically refer to mistreatment due to adolescents’ race/ethnicity. One additional item was added (“Did people act suspicious of you because of your race/ethnicity?”). A mean score was calculated with higher scores indicating more frequent discrimination. The overall mean of ethnoracial discrimination revealed that reports of discrimination ranged from “*never*” to “*once or twice*” (i.e., *M* = 1.53, *SD* = 0.63), which is consistent with means reported in another study with Latino adolescents (Peer *M* = 1.01, *SD* = 1.32, Adult *M* = 0.93, *SD* = 1.30; Saafir et al., 2025,1) as well as an ethnoracially diverse sample including Latino adolescents (e.g., Peer *M* = 1.78, *SD* = 0.82, School *M* = 1.31, *SD* = 0.60; Montoro et al., 2021). Prior work including Latino youth has demonstrated strong internal consistency and supported the validity of the measure (Douglass & Umaña-Taylor, 2017). In the current study, Cronbach’s α = 0.89.

#### Behavioral and Emotional Academic Engagement (T2)

Adolescents’ levels of behavioral and emotional engagement with school were assessed using the Engagement versus Disaffection with Learning: Student Report Scale (Skinner et al., 2008). The behavioral subscale consists of six items (e.g., “I try hard to do well in school”), and the emotional subscale includes four items (e.g., “When I am in class, I feel good”). Items were scored on a 5-point Likert scale (0 = *never* to 4 = *all the time*). Both subscales have demonstrated adequate reliability and support for validity with Latino adolescents (Wantchekon & Umaña-Taylor, 2021). The current study examined students’ behavioral and emotional engagement as unique constructs by averaging each set of items, such that higher scores reflected greater engagement. Cronbach’s α were = 0.83 and α = 0.84 for behavioral and emotional engagement, respectively.

#### Academic Self-Efficacy (T2)

Adolescents were asked to report their perceived competence to complete their class work using the self-efficacy scale of the Patterns of Adaptive Learning Scales (Midgley et al., 2000). The scale consists of five items (e.g., “I’m certain I can master the skills taught in class this year”) scored on a 5-point Likert scale (1 = *not at all true* to 5 = *very true*). Prior work has supported the reliability and validity of this scale with Latino youth (Zychinski & Polo, 2012). Mean scores were computed and higher values reflected higher academic self-efficacy; Cronbach’s α = 0.92.

#### School Belonging (T2) 

Adolescents completed the school belonging scale (McNeely et al., 2002), which consists of five items (e.g., “I feel like I am part of this school”) that were scored on a 5-point Likert scale (1 = *strongly disagree* to 5 = *strongly agree*). Prior work with Latino youth has provided support for adequate reliability and validity for this scale (Fernandez et al., 2019). A mean composite score was computed, and higher scores reflected a stronger sense of school belonging. In the current study, Cronbach’s α = 0.86.

#### Covariates

Students were asked to report their gender (recoded as a dichotomous variable with female, non-binary or other gender = 0 and male = 1) and grade level (9th grade to 12th grade). Given prior work noting variability in cultural wealth and academic outcomes by these sociodemographic characteristics (e.g., Brass et al., 2019; Cupito et al., 2015, gender and grade were included as covariates.

### Analytic Approach

Preliminary analyses included examining descriptive statistics (e.g., means, correlations), as well as conducting attrition analyses to assess whether participating and non-participating youth differed on study variables. Following these descriptives analyses, latent profile analyses (LPA) using Mplus v. 8.7 (Muthén & Muthén, 2023) were carried out to examine patterns of adolescents’ reports of FES, ERI, and emotional support from Latino and non-Latino friends. Analyses used the full information maximum likelihood estimator with robust standard errors (i.e., MLR; Muthén & Muthén, 2023), and cases that were missing data on all profile indicators were removed from analyses (Ferguson et al., 2020). Little’s MCAR (i.e., missing completely at random) test was not statistically significant (χ^2^ (205) = 212.75, *p* = .34). Models were estimated until model fit decreased. Model fit was assessed using Akaike Information Criteria (AIC) and sample-size adjusted Bayesian Information Criterion (ABIC) values with lower values indicating better fit (Schwarz, 1978). The Lo-Mendell-Rubin test (LMRT; Lo et al., 2001) was also considered; this compares model fit between *k* and *k* – 1 profile solutions and a *p* value of less than 0.05 indicates that the current profile solution (*k*) is significantly better compared to the *k* – 1 solution. The selection of the final profile solution was based on a combination of fit statistics, as well as the conceptual interpretability of profiles (Collins & Lanza, 2009). After determining the final profile solution, an omnibus Wald test was conducted to estimate whether profiles differed on cultural wealth indicators, followed by pairwise mean comparisons when the omnibus test was statistically significant. As previously described, profile labels with respect to ERI exploration and resolution were guided by Marcia’s operationalization of Erikson’s psychosocial development theory (1968), which was later adapted by Phinney (Phinney & Ong, 2007) and Umaña-Taylor and colleagues (2004) to capture distinct statuses of youth’s’ ERI development (e.g., achieved, diffused). Profile labels specific to Latino and non-Latino friend support were also created to capture whether friend support was comparably high (i.e., High Friend Support) across Latino and non-Latino friends, whether levels were low across both friendship networks (i.e., Low Friend Support), or whether peer support was more mixed such that one source of peer support was higher/lower relative to the other source (i.e., Mixed Friend Support).

Next, the R3-step approach in Mplus v. 8.7 (Asparouhov & Muthén, 2019) was used to examine whether classification into profiles was predicted by adolescents’ gender and grade level. To examine differences on academic adjustment, the Bolck, Croon, and Hagenaars (BCH) method (Bolck et al., 2004) was employed. This method uses weights to fix class membership, thus preventing class shifts, and accounts for measurement error (Asparouhov & Muthén, 2019). To evaluate whether academic adjustment means varied by profile membership, a series of Wald tests were performed. A significant omnibus Wald test indicated that means were not equivalent across profiles, and pairwise comparisons were then used to identify differences. Finally, to examine whether profile membership moderated the association between discrimination and academic adjustment, multigroup BCH analyses (with profile membership as the grouping variable) were conducted.

For all analyses, effect sizes were also estimated and evaluated. To compare pairs of profile means on cultural wealth and academic adjustment indicators, *d* values were calculated by dividing mean differences between profile pairs by the common standard deviation (i.e., residual variances were constrained equal; L. Muthen, personal communication, November 4th, 2025); *d* values that were 0.20 or lower were considered small, 0.50 was medium, and 0.80 was large (Cohen, 1988). For moderation analyses, *R*^2^ values were reported to capture the proportion of variance explained. To evaluate *R*^*2*^ values for moderation analyses, values of 0.02, 0.13, and 0.26 were considered small, medium, and large, respectively (Cohen, 1988).

## Results

### Descriptive Characteristics

Means, correlations, and other descriptive statistics are presented in Table 1. Attrition analyses to examine whether adolescents who participated at T2 differed from non-participating adolescents on T1 study variables indicated that participating (*n* = 467) and non-participating (*n* = 160) adolescents did not differ on FES, *t*(603) = − 0.99, *p* = .32, ERI exploration, *t*(618) = -1.21, *p* = .23, ERI resolution *t*(619) = − 0.97, *p* = .33, Latino friend support *t*(238) = − 0.64, *p* = .52, or non-Latino friend support *t*(255) = -1.76, *p* = .08. Furthermore, no differences emerged due to adolescents’ gender χ^2^(1) = 1.14, *p* = .29, or grade level *t*(619) = 0.19, *p* = .84.

### Profiles of Cultural Wealth among Latino Adolescents

**Fig. 1:**
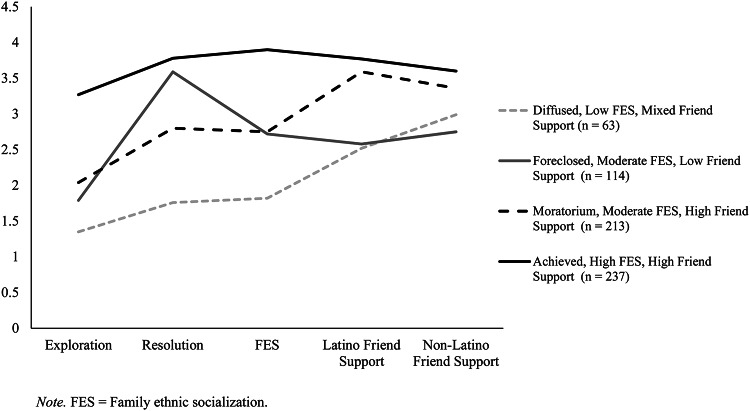
Final profile solution comprised of Latino adolescents’ cultural wealth indicators

Table 2 presents the 1- to 7-profile LPA fit statistics. Based on the decreasing AIC, ABIC, and LMRT values, each subsequent profile solution fit better than the prior one with the exception of the 7-profile solution. Each of the first six solutions were plotted and compared to better understand the interpretability of profiles. A closer look at the 5- and 6-profile solutions revealed that some profiles were not distinct from one another in meaningful ways. For instance, the 5-profile solution consisted of one small profile (*n* = 89) of adolescents who reported slightly lower levels of cultural wealth compared to a larger profile (*n* = 233; see online materials). Similarly, the 6-profile solution consisted of smaller profiles that did not demonstrate a unique pattern from larger profiles in the solution. In contrast, the 4-profile solution demonstrated distinct profiles that were conceptually different from one another. Given these considerations, the 4-profile solution was selected as the final model (see Fig. 1).

**Table 1:** Correlations, means, and standard deviations for all study variables

Variable	1	2	3	4	5	6	7	8	9	10	11	12
1. T1 Grade												
2. T1 Gender	-0.07											
3.T1 Exp	0.19***	-0.19***										
4. T1 Res	0.14***	-0.15***	0.55***									
5. T1 FES	0.11**	− 0.16***	0.65***	0.55***								
6. T1 Latino	0.09	-0.30***	0.29***	0.16*	0.27***							
7. T1 N-Latino	0.14*	-0.26***	0.23***	0.08	0.21***	0.78***						
8. T1 Disc	0.16***	0.05	0.23***	0.15***	0.26***	0.09	0.21***					
9. T2 Behave	0.08	− 0.13**	0.14*	0.19***	0.21***	0.19*	0.18*	− 0.01				
10. T2 Emotion	0.15**	0.01	0.14*	0.16***	0.14**	0.16*	0.16*	0.001	0.67***			
11. T2 Efficacy	0.02	-0.10*	0.11*	0.14**	0.15**	0.12	0.15	− 0.10	0.49***	0.47***		
12. T2 Belong	− 0.04	0.07	0.10*	0.10*	0.07	0.11	0.08	− 0.20***	0.37***	0.44***	0.40***	
*M*	10.26	0.41	2.39	3.21	3.09	3.41	3.30	1.53	2.48	2.07	3.61	3.35
*SD*	1.10	0.49	0.91	0.75	0.97	0.98	0.97	0.63	0.67	0.78	0.83	0.80
Range	9–12	0–1	1–4	1–4	1–5	1–5	1–5	1–5	0–4	0–4	1–5	1–5

Note. *N* = 627 adolescents. T1 = Time (1) T2 = Time (2) Gender coded as 0 = female, other; 1 = male. Exp =Exploration. Res = Resolution. FES = Family ethnic socialization. Latino = Latino friend emotional support. N-Latino = Non-Latino friend emotional support. Disc = ethnic-racial discrimination. Behave = Behavioral academic engagement. Emotion = Emotional academic engagement. Efficacy = Academic Efficacy. Belong = School Belonging. * *p* < .05. ** *p* < .01. *** *p* < .001

**Table 2:** Fit statistics for latent profile analysis among latino students using family ethnic socialization, ethnic-racial identity exploration and resolution, emotional support from latino friends, and emotional support from non-latino friends

	1-profile	2-profile	3-profile	4-profile	5-profile	6-profile	7-profile
AIC	6146.71	5611.20	5446.45	5391.23	5285.08	5219.68	5197.54
ABIC	6159.37	5631.46	5474.31	5426.68	5328.12	5270.33	5255.78
LMRT *p* value		*p* < .001	*p* < .001	*p* = .04	*p* < .001	*p* < .001	*p = .*23
Entropy		0.77	0.80	0.77	0.89	0.86	0.84
Smallest class (% of sample)		289 (46%)	71 (11%)	63 (10%)	29 (5%)	29 (5%)	29 (5%)

Note. *N* = 627 Latino adolescents. AIC = Akaike Information Criterion; ABIC = Adjusted Bayesian Information Criterion; LMRT = Lo-Mendell-Rubin Adjusted Likelihood Ratio Test

Table 3a presents the means for the 4-profile solution. Profile labels were based on substantive differences that emerged among profiles, whether friend support was consistently high/low across Latino and non-Latino friend networks, as well as prior conceptual work pertaining to ERI status. Youth in the largest profile (*n* = 237; 38% of the sample) reported the highest levels of exploration, resolution, FES, and Latino friend and non-Latino friend support. Given that exploration (*M* = 3.27) and resolution (*M* = 3.78) levels for this profile were higher relative to all other profiles, as evident by the Wald tests (see Table 3a) and the large effect sizes among pairwise mean comparisons (e.g., *d*_*range*_ = 2.21–3.44; see online materials), as well as prior work that has labeled similar levels of exploration and resolution as “high” (e.g., Romero et al., 2017; Toomey et al., 2013), this profile was considered to capture youth in an *Achieved* status. Furthermore, Wald test comparisons revealed relatively higher levels of friend support from both Latino (*d*_range_ = 1.40–1.45) and non-Latino (*d*_range_ = 0.66-0.92) friends compared to two other profiles, along with more frequent FES experiences (*d*_range_ = 1.69–3.06) compared to all other profiles. Thus, this profile was labeled *Achieved ERI*,* High FES*,* High Friend Support.*

In contrast, the smallest profile (*n* = 63; 10% of sample) exhibited significantly lower levels of exploration, resolution, and FES compared to all other profiles (Table 3a), which was also evident in effect size differences (see online materials). Notably, exploration (*M* = 1.35) and resolution (*M* = 1.76) levels were also lower than those reported in previous work that designated profiles as being in a “Diffused” status (e.g., exploration *M* = 2.22, resolution *M* = 2.43: Douglass & Umaña-Taylor, 2015b). Pairwise Wald tests also indicated that this profile exhibited low Latino friend support (Table 3a), and non-Latino friend support was moderate and not significantly different from the lowest or highest non-Latino friend support means (Table 3a; *d*_range_ = 0.26-0.66). This profile was labeled *Diffused*,* Low FES*,* Mixed Friend Support*.

A third profile characterizing 18% of the sample (*n* = 114) exhibited low levels of exploration (which was moderately higher than the *Diffused* profile, *d* = 0.78), high levels of resolution, and moderate levels of FES relative to other profiles. Moreover, exploration levels (*M* = 1.79) for this profile were lower than means that have emerged in prior studies with a “Foreclosed” status (e.g., exploration *M* = 2.63; Toomey et al., 2013). Resolution (*M* = 3.59) levels were comparable to the means reported for profiles characterized as “Foreclosed” in prior studies (e.g., *M* = 3.48; Toomey et al., 2013). Latino friend support was low and did not significantly differ from the mean in the profile with the lowest level of Latino friend support (i.e., the *Diffused* profile, *d* = 0.07). Furthermore, this profile also reported the lowest non-Latino friend support. This profile was labeled *Foreclosed*,* Moderate FES*,* Low Friend Support.*

Finally, the fourth profile, which comprised 213 adolescents (34% of the sample), reported higher exploration (*M* = 2.04) compared to the *Diffused*,* Low FES*,* Mixed Friend Support* profile (*M* = 1.35), reflecting a large effect size (*d* = 1.23); this group also reported moderately higher exploration than those in the *Foreclosed*,* Moderate FES*,* Low Friend Support* profile (*M* = 1.79), reflecting a small-to-medium effect size, (*d* = 0.44). Furthermore, this profile exhibited significantly lower levels of resolution (*M* = 2.80) than the *Foreclosed*,* Moderate FES*,* Low Friend Support* profile (*M* = 3.59) and the *Achieved*,* High FES*,* High Friend Support* profile (*M* = 3.78), with differences reflecting large effect sizes (*d* = 2.59 and *d* = 2.09, respectively). This profile also was characterized by moderate levels of FES and Latino and non-Latino friend support means that did not differ from the profiles with the highest levels of friend support. To capture the variability evidenced across these cultural wealth indicators, this profile was labeled *Moratorium*,* Moderate FES*,* High Friend Support*. The reference to the ERI status of Moratorium in this profile label, which reflects higher ERI exploration and lower ERI resolution, is consistent with mean levels of resolution characterized as “low” in prior studies (e.g., resolution *M* = 2.70, Wantchekon & Umaña-Taylor, 2021). Though the exploration mean for this profile is relatively lower than the levels of exploration that have been classified as “high” in prior studies (e.g., exploration *M* = 2.93 Wantchekon & Umaña-Taylor, 2021), in the current study this level of exploration is significantly higher, by a large margin (i.e., large effect size noted above), than the levels of exploration for those in the *Diffused* and *Foreclosed* profiles.

Findings from the R3-step procedure indicated that adolescents in lower grades were more likely to be members of the *Diffused*,* Low FES*,* Mixed Friend Support* (*B* = − 0.38, *SE* = 0.16, *p* = .02) and the *Moratorium*,* Moderate FES*,* High Friend Support (B* = − 0.25, *SE* = 0.11, *p* = .02) profiles relative to the *Achieved* profile. Gender did not predict profile membership.

**Table 3:** Estimated means for (a) cultural wealth indicators and (b) academic adjustment indicators for the 4-profile solution

Omnibus Wald Tests		Diffused, Low FES, Mixed Friend Support (*n* = 63)	Foreclosed, Moderate FES, Low Friend Support (*n* = 114)	Moratorium, Moderate FES, High Friend Support (*n* = 213)	Achieved, High FES, High Friend Support (*n* = 237)
**(a) Cultural Wealth Indicators**					
ERI exploration	χ^2^(3) = 551.46, *p* < .001	1.35 (0.08)^a^	1.79(0.12)^b^	2.04 (0.07)^b^	3.27 (0.06)^c^
ERI resolution	χ^2^(3) = 417.73, *p* < .001	1.76 (0.11)^a^	3.59 (0.10)^b^	2.80 (0.07)^c^	3.78 (0.03)^d^
FES	χ^2^(3) = 444.68, *p* < .001	1.82 (0.11)^a^	2.72 (0.15)^b^	2.75 (0.09)^b^	3.90 (0.05)^c^
L support	χ^2^(3) = 28.32, *p* < .001	2.52 (0.32)^a^	2.58 (0.23)^a^	3.59 (0.15)^b^	3.77 (0.10)^b^
NL support	χ^2^(3) = 12.99, *p =* .005	2.99 (0.31)^a,b,c^	2.75 (0.22)^c^	3.36 (0.13)^b,d^	3.60 (0.11)^a,d^
**(b) Academic Adjustment Indicators**					
Behavioral Eng	χ^2^(3) = 11.87, *p = .*01	1.98 (0.29)^a,c^	2.25 (0.31)^b,c,d^	2.12 (0.31)^a,b^	2.36 (0.31)^d^
Emotional Eng	χ^2^(3) = 12.49, *p* = .01	0.87 (0.35)^a,d^	1.18 (0.37)^c,d,e^	1.21 (0.37)^a,b,c^	1.42 (0.37)^b,e^
Academic Efficacy	χ^2^(3) = 8.97, *p* = .03	3.41 (0.38)^a,c^	3.69 (0.39)^b,c,d^	3.58 (0.38)^a,b^	3.83 (0.39)^d^
School Belonging	χ^2^(3) = 12.47, *p* = .01	3.64 (0.40)^a,c^	3.89 (0.43)^b,c,d^	3.92 (0.42)^a,b^	4.16 (0.42)^d^

Note. *N *= 627 Latino adolescents. FES = Family ethnic socialization. ERI = Ethnic-racial identity. L = Latino. NL = Non-Latino. Behavioral Eng = Behavioral engagement; Emotional Eng= emotional engagement. Means of academic adjustment were estimated after accounting for adolescents’ grade and gender. Within rows, means that do not have the same superscript are significantly different from one another at *p* < .05

### Profile Membership and Academic Outcomes

Table 3b presents profile differences on behavioral engagement, emotional engagement, academic efficacy, and school belonging. Across all academic adjustment indicators, Wald’s omnibus tests indicated differences across profiles. Pairwise Wald’s tests revealed the same pattern of profile differences for three of the four academic adjustment outcomes. Specifically, adolescents in the *Achieved*,* High FES*,* High Friend Support* profile reported higher behavioral engagement, academic efficacy, and school belonging compared to adolescents in the *Diffused*,* Low FES*,* Mixed Friend Support* profile (Wald χ^2^(1) = 7.96, *p* = .005, *d* = 0.55; Wald χ^2^(1) = 6.61, *p* = .01, *d =* 0.51; Wald χ^2^(1) = 10.10, *p* = .002. *d =* 0.65, respectively) and the *Moratorium*,* Moderate FES*,* High Friend Support* profile (Wald χ^2^(1) = 6.76, *p* = .0, *d =* 0.36; Wald χ^2^(1) = 4.16, *p* = .01, *d =* 0.29; Wald χ^2^(1) = 4.60, *p* = .03, *d =* 0.30), but not the *Foreclosed*,* Moderate FES*,* Low Friend Support* profile (Wald χ^2^(1) = 0.91, *p* = .34, *d =* 0.17; Wald χ^2^(1) = 0.92, *p* = .34 , *d =* 0.17; Wald χ^2^(1) = 3.01, *p* = .08, *d =* 0.32, respectively). Thus, all effect sizes comparing means between the *Achieved*,* High FES*,* High Friend Support* and the *Diffused*,* Low FES*,* Mixed Friend Support* profile, as well as the *Moratorium*,* Moderate FES*,* High Friend Support* profiles were small to medium, but when compared to the *Foreclosed*,* Moderate FES*,* Low Friend Support* profile, mean differences were generally small in magnitude. Adolescents in the *Achieved*,* High FES*,* High Friend Support* profile also reported greater emotional engagement relative to the *Diffused*,* Low FES*,* Mixed Friend Support* profile (Wald χ^2^(1) = 10.83, *p* = .001, *d =* 0.67), but adolescents in the *Achieved* profile did not significantly differ from the *Foreclosed*,* Moderate FES*,* Low Friend Support* (Wald χ^2^(1) = 3.21, *p* = .07, *d =* 0.28) or the *Moratorium*,* Moderate FES*,* High Friend Support* (Wald χ^2^(1) = 3.96, *p* = .05, *d =* 0.31) profiles, both of which demonstrated small-to-medium effect sizes.

### Ethnoracial Discrimination and Academic Adjustment: Moderation by Profile Membership

To examine the extent to which associations between discrimination and academic adjustment were moderated by profile membership, multigroup BCH models were carried out (with profile membership as the grouping variable) for each outcome separately. The omnibus Wald test for the association between adolescents’ T1 ethnoracial discrimination and T2 behavioral academic engagement was statistically significant (Wald χ^2^(3) = 10.27, *p* = .02; Table 4). When examining differences between profiles, more frequent T1 ethnoracial discrimination was associated with greater T2 behavioral engagement for the *Diffused*,* Low FES*,* Mixed Friend Support* profile (*B* = 0.50, *SE* = 0.23, *p* = .03; *R*^*2*^ = 0.22). In contrast, more T1 ethnoracial discrimination was associated with lower T2 behavioral engagement for the *Moratorium*,* Moderate FES*,* High Friend Support* (*B* = − 0.41, *SE* = 0.16, *p* = .01, *R*^*2*^ = 0.16; χ^2^(1) = 9.44, *p* = .002) profile, and T1 ethnoracial discrimination and T2 behavioral engagement were not significantly associated for the *Achieved*,* High FES*,* High Friend Support* profile (*B* = − 0.05, *SE* = 0.09, *p* = .57, *R*^*2*^ = 0.01; χ^2^(1) = 4.98, *p* = .03). The association was also not statistically significant for the *Foreclosed*,* Moderate FES*,* Low Friend Support* profile (*B* = 0.50, *SE* = 0.30, *p* = .09, *R*^*2*^ = 0.09), and pairwise tests revealed that this profile was significantly different from the *Moratorium*,* Moderate FES*,* High Friend Support* profile (χ^2^(1) = 4.10, *p* = .04). No other differences emerged.

**Table 4:** Unstandardized estimates for the association between discrimination and academic adjustment among the 4-Profile solution

T1 Discrimination to T2 Academic Adjustment Outcomes	Omnibus Wald Test	Diffused, Low FES, Mixed Friend Support (*n* = 63)	Foreclosed, Moderate FES, Low Friend Support (*n* = 114)	Moratorium, Moderate FES, High Friend Support (*n* = 213)	Achieved, High FES, High Friend Support (*n* = 237)
Disc to Behavioral Engagement	χ^2^(3) = 10.27, *p =* .02	0.50 (0.23)*^b^	0.50 (0.30)^b,c^	− 0.41 (0.16)*^a^	− 0.05 (0.09)^a,c^
Disc to Emotional Engagement	χ^2^(3) = 12.77, *p* = .01	0.18 (0.33)^a,b^	0.27 (0.34)^b,c^	− 0.54 (0.13)***^d^	0.04 (0.10)^a,c^
Disc to Academic Efficacy	χ^2^(3) = 10.52, *p* = .01	0.32 (0.25)^c^	− 0.66 (0.37)^b,d^	− 0.44 (0.17)*^a,b^	− 0.08 (0.09)^a,c,d^
Disc to School Belonging	χ^2^(3) = 5.05, *p* = .17	− 0.33 (0.08)***^a^	− 0.33 (0.08)***^a^	− 0.33 (0.08)***^a^	− 0.33 (0.08)***^a^

Note. *N* = 627 Latino adolescents. Unstandardized estimates (standard errors) presented. Disc = Ethnoracial discrimination. FES = Family ethnic socialization. Coefficients were estimated after accounting for adolescents’ gender and grade level. Within rows, means that do not have the same superscript are significantly different from one another at *p* < .05. *p < .05. *** *p* < .001

Turning to emotional academic engagement, the omnibus Wald test was statistically significant (Wald χ^2^(3) = 12.77, *p* = .01), indicating that the association varied by profile membership. More frequent T1 ethnoracial discrimination was associated with adolescents’ lower T2 emotional academic engagement for the *Moratorium*,* Moderate FES*,* High Friend Support* profile (*B* = − 0.54, *SE* = 0.13, *p* < .01, *R*^*2*^ = 0.19). The *Moratorium* profile was significantly different from the other three profiles: *Achieved*,* High FES*,* High Friend Support* profile (*B* = 0.04, *SE* = 0.10, *p* = .67, *R*^*2*^ = 0.01; χ^2^(1) = 11.88, *p* = .001), *Foreclosed*,* Moderate FES*,* Low Friend Support* profile (*B* = 0.27, *SE* = 0.34, *p* = .43, *R*^*2*^ = 0.03; χ^2^(1) = 4.10, *p* = .04), and the *Diffused*,* Low FES*,* Mixed Friend Support* profile (*B* = 0.18, *SE* = 0.33, *p* = .58; *R*^*2*^ = 0.04; χ^2^(1) = 3.85, *p* = .04). Moreover, the remaining profiles had nonsignificant associations that did not differ from each other.

The omnibus Wald test for the association between discrimination and academic efficacy was statistically significant (Wald χ^2^(3) = 10.52, *p* = .01), indicating that associations varied across profiles. When examining differences between profiles, more frequent T1 ethnoracial discrimination was associated with lower T2 academic efficacy for the *Moratorium*,* Moderate FES*,* High Friend Support* profile (*B* = − 0.44, *SE* = 0.17, *p* = .01; *R*^*2*^ = 0.13), and this association was significantly different from the nonsignificant association that emerged for the *Diffused*,* Low FES*,* Mixed Friend Support* profile (*B* = 0.32, *SE* = 0.25, *p* = .20, *R*^*2*^ = 0.07; χ^2^(1) = 6.24, *p* = .01) profile. The *Diffused*,* Low FES*,* Mixed Friend Support* profile was also statistically different from the *Foreclosed*,* Moderate FES*,* Low Friend Support* (*B* = − 0.66, *SE* = 0.37, *p* = .07, *R*^*2*^ = 0.10; χ^2^(1) = 4.94, *p* = .03) profile, although both associations were nonsignificant. No other differences emerged.

Finally, the omnibus Wald test examining the association between discrimination and school belonging was not statistically significant (Wald χ^2^(3) = 5.05, *p* = .17), indicating that the association did not vary across the profiles. Thus, paths were constrained to be equal across profiles, and results indicated that for all profiles, higher T1 ethnoracial discrimination was associated with lower T2 school belonging (*B* = − 0.33, *SE* = 0.08, *p* = .001; *R*^*2*^ = 0.11).

## Discussion

Despite being one of the fastest growing ethnoracial groups in the U.S., Latino youth continue to be underrepresented in higher education institutions (National Center for Education Statistics, 2024), which could have detrimental effects on their future life chances. Previous work assessing cultural wealth, ethnoracial discrimination, and youth's academic adjustment has typically employed a variable-centered approach. Latino youth, however, may not experience cultural wealth in a uniform way, and different configurations and patterns of cultural wealth may differentially relate to academic adjustment. Indeed, the present findings extend prior work by revealing subgroups of Latino adolescents who varied in their combination of cultural wealth indicators. Furthermore, youth with more cultural wealth reported greater academic adjustment relative to youth with more varied sources. In addition, adolescents in the profile with more moderate ERI and FES components and high friend support (i.e., *Moratorium*,* Moderate FES*,* High Friend Support*) emerged as the most vulnerable to experiences of ethnoracial discrimination, demonstrating that having high levels of friend support may not fully compensate for more moderate levels of other sources of cultural wealth.

### Profiles of Latino Adolescents’ Cultural Wealth and Implications for Academic Adjustment

Grounded in the community cultural wealth model (Yosso, 2005), this study examined potential heterogeneity in adolescents’ cultural wealth as indicated by their FES, ERI, and emotional support from Latino and non-Latino friends. Indeed, profiles ranged from relatively low (e.g., *Diffused*,* Low FES*,* Mixed Friend Support*) to mostly moderate (i.e., *Moratorium*,* Moderate FES*,* High Friend Support*) and high levels of cultural wealth (i.e., *Achieved*,* High FES*,* High Friend Support*), as well as one profile that had more variable cultural wealth (i.e., *Foreclosure*,* Moderate FES*,* Low Friend Support*). Thus, findings revealed that adolescents were at different statuses of ERI development, which has also been found in prior LPA studies with Latino adolescents (e.g., Wantchekon & Umaña-Taylor, 2021). In the current study, the *Achieved*,* High FES*,* High Friend Support* profile was the largest profile, comprising over a third of the sample (38%). The presence and size of this profile suggests that many Latino adolescents were progressing in their ERI development with high levels of cultural wealth. Furthermore, adolescents in more advanced grades were more likely to be classified in the *Achieved*,* High FES*,* High Friend Support* profile compared to the *Moratorium*, and the *Diffused* profiles. Consistent with theory, as adolescents advance in grade level, their greater sense of autonomy may enable more exploration resulting in clarity about their ERI, and they may have more time to develop close bonds with Latino and non-Latino friends and have more experience learning about their cultural background with their family (Umaña-Taylor et al., 2009; Way & Silverman, 2012).

Among the remaining profiles, the *Diffused*,* Low FES*,* Mixed Friend Support* profile was the smallest (10%) subgroup and consisted of adolescents who reported the lowest levels of cultural wealth, except for non-Latino friend support. Interestingly, the *Moratorium*,* Moderate FES*,* High Friend Support* profile included adolescents who had engaged in relatively high levels of exploration, particularly when compared to youth in the *Diffused* and *Foreclosed* profiles, relatively low resolution regarding their ERI, had moderate levels of FES experiences, and reported some of the highest levels of friend emotional support. Thus, findings may have illuminated a subgroup of adolescents in a state of identity confusion and rumination such that they have been unable to attain a sense of clarity regarding how their ethnic-racial background informs their understanding of themselves, despite evidence of some exploration of this identity domain (Phinney, 1993; Phinney & Ong, 2007). Furthermore, given the high level of Latino and non-Latino friend support, youth in this profile may have been prioritizing their friendships over gaining clarity about their ERI. Overall, findings elucidated the value of using person-centered approaches to better understand Latino adolescents’ heterogenous experiences with various sources of cultural wealth.

In partial support of the second hypothesis, adolescents in the *Achieved*,* High FES*,* High Friend Support* profile reported better academic adjustment relative to profiles with more variability in cultural wealth, although findings differed by outcomes. Specifically, Wald tests and small-to-medium effect sizes demonstrated that adolescents in the *Achieved*,* High FES*,* High Friend Support* profile reported better academic adjustment compared to the *Diffused*,* Low FES*,* Mixed Friend Support* profile across all academic outcomes, and as compared to the *Moratorium*,* Moderate FES*,* High Friend Support* profile across all outcomes except for emotional engagement. These differences between profiles in a more advanced ERI status and those that are possibly in a status of identity confusion or rumination resemble findings from another person-centered study including Latino adolescents, which revealed that those with a more developed ERI (and higher ERI positive affect) reported greater behavioral and emotional academic engagement compared to the subgroups with a Diffused ERI status as well as positive and negative affect (Wantchekon & Umaña-Taylor, 2021). Moreover, the mean scores of school belonging for the *Diffused*,* Low FES*,* Mixed Friend Support* profile were relatively lower than the school belonging means in previous work with Latino early adolescents that used the same measure and response scale, whereas the means for the *Achieved*,* High FES*,* High Friend Support* profile were relatively higher, suggesting that cultural wealth dimensions are informative for understanding how Latino youth are navigating their academic context (Medina et al., 2020). In terms of practical implications, findings suggest that Latino adolescents with more cultural wealth may be more self-assured in their ability to be academically successful and feel a greater sense of connection to their school environment, which in turn may be promotive of long-term academic achievement. On the other hand, Latino adolescents with more limited support and in a less developed identity status may be having more difficulty navigating their academic contexts and thus greater attention is needed to identify these youth and explore whether more opportunities to develop cultural wealth or other strategies are needed to promote their academic adjustment.

Interestingly, the academic outcomes exhibited by the *Diffused*,* Low FES*,* Mixed Friend Support* profile revealed that having high levels of non-Latino friend support may not offer compensatory benefits if adolescents have low levels of familial and navigational capital. Indeed, the *Foreclosed*,* Moderate FES*,* Low Friend Support* profile reported similar levels of academic adjustment compared to the *Achieved* profile, despite reporting the lowest levels of Latino and non-Latino friend support. Given the limited work assessing profiles consisting of ERI, FES, and friend support, future work may consider additional resources that may be promotive of Latino adolescents’ academic adjustment. For instance, adolescents with a foreclosed identity are typically characterized as relying on the opinions of others when defining their own identity (Marcia, 1980; Umaña-Taylor, 2016b). Thus, other aspects of ERI such as public regard (e.g., how individuals perceive that others evaluate their ethnoracial group; Sellers et al., 1997), may compensate for adolescents’ lower ERI development, which in turn can enable adolescents to feel academically successful (Rivas-Drake et al., 2014). Furthermore, other indicators of familial capital, such as family cohesion, may be important to consider in future work, as previous studies have revealed that spending quality time with family members may compensate for low FES and be promotive of Latino adolescents’ ERI exploration (Constante et al., 2020).

### Cultural Wealth Profiles Mitigate Links between Discrimination and Academic Outcomes

Regarding findings examining how profiles of cultural wealth would protect adolescents from experiences of discrimination, both expected and unexpected results emerged. First, for behavioral academic engagement, findings revealed that among adolescents in the *Diffused*,* Low FES*,* Mixed Friend Support* profile, greater discrimination at the beginning of the academic year was associated with more behavioral academic engagement towards the end of the academic year. It is possible that for youth in the *Diffused* profile, being behaviorally engaged in the classroom is a form of active coping against high levels of ethnoracial discrimination. This form of active coping is typically labeled as a John Henry effect, which refers to cases where individuals facing situational stressors, and without sufficient resources, overcome adversity by overexerting themselves, in turn presenting favorable outcomes in some domains (James, 1994). Adolescents were not assessed on their perceived levels of active coping, thus this explanation is speculative, but represents an important future direction to understand the mechanism through which youth with lower cultural wealth overcome discriminatory experiences. Furthermore, future considerations for understanding adolescents’ academic adjustment may consider the role of adaptive coping strategies (e.g., more direct problem solving; McDermott et al., 2019), particularly among adolescents with lower ERI, FES, and peer support.

Notably, a negative association consistently emerged between ethnoracial discrimination and academic outcomes for the *Moratorium*,* Moderate FES*,* High Friend Support* profile. Despite reporting some of the highest levels of Latino and non-Latino friend support, these adolescents may experience confusion about discrimination, as they have moderately explored their ERI yet have not gained clarity around this identity domain relative to those in the *Foreclosed* and *Achieved* profiles. Specifically, adolescents in the *Moratorium*,* Moderate FES*,* High Friend Support* profile exhibited lower levels of ERI resolution compared to the *Achieved* and *Foreclosed* group, but similar levels of FES as the *Foreclosed* group. Thus, having a more limited understanding regarding their ERI, coupled with moderate levels of exploration may place this subgroup of adolescents at greater risk of identity confusion when they face ethnoracial discrimination (Douglass & Umaña-Taylor, 2017) in an ethnoracially diverse school context, which is not compensated by high friend support. These findings relate to prior work highlighting the psychological benefits conferred by having a sense of confidence regarding one’s ethnoracial group membership (Umaña-Taylor, 2016b). Moreover, moderate engagement in these developmental processes, particularly when resolution is low, may be more detrimental than being completely disengaged, as disengagement may offer some protection due to a complete lack of awareness.

Finally, a robust finding that emerged across all profiles was that more frequent experiences of ethnoracial discrimination were linked with a lower sense of school belonging at the end of the academic year. Prior work has characterized school belonging as an indicator of the interpersonal relationships among students, their peers, and adults in their school (Demanet & Van Houtte, 2012). Thus, Latino adolescents who frequently encounter discriminatory and exclusionary messages from their peers and adults in school feel particularly vulnerable about their acceptance in the school setting (Montoro et al., 2021), and these experiences of ethnoracial marginalization were not compensated for by cultural wealth in the current sample. Drawing from previous studies that examined the role of Latino adolescents’ coping strategies in school settings, future work may consider whether proactive coping (e.g., clarifying misconceptions, being proud of oneself; Montoro et al., 2021) may be protective across subgroups of adolescents in their sense of school belonging. Importantly, it is also possible that Latino adolescents in this particular school setting did not perceive an environment that was culturally affirming, despite the school composition being ethnoracially diverse. Prior work focused on Latino youth suggests that messages from peers and educators that indicate they value Latino students’ cultural background are essential for students to feel like they belong in their school setting (López et al., 2025). As the youth population in the U.S. continues to become more ethnoracially diverse, with Latino students comprising a third of the K-12 population by 2030 (National Center for Education Statistics, 2021), it is critical for educators and school settings more broadly to prevent ethnoracial discrimination in an effort to ensure students’ sense of school belonging is not compromised (Allen et al., 2018).

### Limitations and Future Directions

The current findings make important contributions to the field by revealing that various constellations of community cultural wealth indicators are evident among Latino adolescents and provide novel insights into the promotive and protective effects of profiles. Findings suggest that profiles comprised of more cultural wealth are linked with more favorable academic outcomes; however, most profiles did offer protective effects against ethnoracial discrimination when there was at least one form of cultural wealth (with the exception of school belonging). Despite these strengths, there are limitations worth addressing and additional considerations for future work. First, the current findings identified a subgroup of adolescents who were in somewhat of a moratorium status of ERI development (i.e., moderate exploration, low resolution), but not a clear moratorium status that has emerged in prior work (higher exploration, lower resolution; e.g., Douglass & Umaña-Taylor, 2017). Given that a moratorium status is typically found with younger adolescents (Douglass & Umaña-Taylor, 2017), it is possible that participants in the current study were transitioning and to capture a clearer moratorium status would require a sample that included relatively younger adolescents. Furthermore, there is a limited understanding regarding how ERI typologies work in conjunction with other salient cultural assets to inform Latino adolescents’ academic outcomes. Thus, future work may consider intentional sampling of adolescents at various ERI development statuses to further explore whether and how certain components of cultural wealth (e.g., peer emotional support) compensate for the lack of other resources (e.g., ERI resolution) and inform adolescents’ academic adjustment. Finally, the sample was derived from one school in Arizona, a region where most of the Latino population is of Mexican origin (American Community Survey, 2021), Latinos are a long-term established population, and where anti-Latino and immigrant sentiments and policies are prominent (Ayón, 2016). Given that the Latino population is largely becoming more diverse by national origin group (Moslimani et al., 2023) and residing in diverse regions of the U.S., it will be important to examine these associations in other regions.

## Conclusion

The persistent academic disparities that are experienced by Latino youth suggest that additional work is needed to understand how cultural wealth can positively inform Latino adolescents’ academic adjustment as well as provide a buffer against ethnoracial discrimination experiences. Prior work has primarily used variable-centered approaches that implicitly assume that Latino youth's experiences are homogenous, thereby obscuring the complex ways multiple cultural assets co-occur. Indeed, the current findings revealed four unique configurations of cultural wealth, indicating that Latino adolescents do not experience these sources of cultural wealth in a uniform matter. When examining links between emergent profiles and academic adjustment, the subgroup of adolescents who exhibited the highest levels of cultural wealth reported better academic adjustment relative to the subgroup of adolescents who reported low-to-moderate experiences with cultural wealth. Thus, learning about one’s cultural background (through one’s own exploration and familial experiences), feeling more confident in understanding one’s ethnic-racial identity, and establishing emotionally supportive friendships with peers from culturally similar and distinct backgrounds are salient and valuable sources of cultural wealth that may enable Latino adolescents to be better academically adjusted. When examining whether profile membership moderated links between ethnoracial discrimination and academic adjustment, the profile that emerged as most vulnerable to ethnoracial discrimination was not the subgroup of adolescents with the lowest levels of cultural wealth, but rather another profile with moderate experiences learning about their ethnoracial background, low levels of resolution, and high levels of friend support. These findings suggest that Latino adolescents who may be most at risk are those who have started the process of learning about their ethnoracial background but may have not had sufficient opportunities to further inquire and gain a sense of clarity about what their ethnic-racial identity means to them. Furthermore, all youth who experienced more frequent ethnoracial discrimination exhibited lower school belonging, regardless of variation in their sources of cultural wealth. Adolescence is a critical developmental period during which youth are engaging in the normative developmental process of identity development, seeking positive familial and peer relationships, and navigating a system of ethnoracial stratification that makes them susceptible to experiencing discrimination. Thus, it is imperative for future efforts that address academic disparities among Latino youth to assess risk factors alongside cultural assets. Furthermore, findings from the current study suggest that an important avenue will be to account for Latino adolescents’ diverse experiences, as some adolescents may be more socially and psychologically equipped to navigate their educational contexts than others.

## Supplementary Information

Below is the link to the electronic supplementary material.

Supplementary Material 1

## Data Availability

The datasets generated and/or analyzed during the current study are not publicly available but are available from the corresponding author on reasonable request.

## Footnotes

1 Saafir and colleagues used the following Likert scale: 0 = never, 1 = once or twice, 2 = a few times, 3 = a lot, and 4 = a whole lot).
