# Supplementary material for: A Latent Profile Analysis of Latino Adolescents’ Cultural Wealth, Ethnic-Racial Discrimination, and Academic Adjustment
Source: J Youth Adolesc. 2026 Feb 19;55(6):1472–88. doi: 10.1007/s10964-026-02328-7 (PMC13263244; doi:10.1007/s10964-026-02328-7)
Supplement: Supplementary file 1 — Supplementary Material 1 [file 10964_2026_2328_MOESM1_ESM.docx]

|  | |  |  |  |  |  |
| --- | --- | --- | --- | --- | --- | --- |
| **Table S1**  *Effect Sizes for Pairwise Mean Comparisons of Cultural Capital Indicators* | | | | | | |
|  | | **Effect Size by Cultural Capital Indicator** | | | | |
| Pairwise Profile Comparisons | | *Exploration* | *Resolution* | *Family Ethnic Socialization* | *Latino Friend Support* | *Non-Latino Friend Support* |
| **Achieved, High FES, High Friend Support (comparison group)** | | | | | |  |
| Moratorium, Moderate FES, High Friend Support | | 2.21 | 2.59 | 1.69 | .21 | .25 |
| Foreclosed, Moderate FES, Low Friend Support | | 2.65 | .51 | 1.73 | 1.40 | .92 |
| Diffused, Low FES, and Mixed Friend Support | | 3.44 | 5.35 | 3.06 | 1.45 | .66 |
| **Moratorium, Moderate FES, High Friend Support (comparison group)** | | | | |  |  |
| Foreclosed, Moderate FES, Low Friend Support | | .44 | 2.09 | .04 | 1.19 | .67 |
| Diffused, Low FES, and Mixed Friend Support | | 1.23 | 2.76 | 1.37 | 1.26 | .40 |
| **Foreclosed, Moderate FES, Low Friend Support (comparison group)** | | | | |  |  |
| Diffused, Low FES, and Mixed Friend Support | | .78 | 4.84 | 1.33 | .07 | .26 |
| *Note.* FES = Family ethnic socialization. Effect sizes were estimated by calculating the mean difference between two profile means for each indicator, and dividing by the common standard deviation. To interpret effect sizes, values that were .20 or lower were considered small, .50 was medium, and .80 was large (Cohen, 1988). | | | | | | |

**Figure S1.**

*Two-profile solution comprised of Latino adolescents’ cultural capital*

*Note.* FES = Family ethnic socialization.

**Figure S2.**

*Three-profile solution comprised of Latino adolescents’ cultural capital.*

*Note.* FES = Family ethnic socialization.

**Figure S3.**

*Five-profile solution comprised of Latino adolescents’ cultural capital.*

*Note.* FES = Family ethnic socialization.

**Figure S4.**

*Six-profile solution comprised of Latino adolescents’ cultural capital.*

*Note.* FES = Family ethnic socialization.
